# Neighborhood greenspace and risk of type 2 diabetes in a prospective cohort: the Multi-Ethncity Study of Atherosclerosis

**DOI:** 10.1186/s12940-021-00824-w

**Published:** 2022-01-16

**Authors:** Annie Doubleday, Catherine J. Knott, Marnie F. Hazlehurst, Alain G. Bertoni, Joel D. Kaufman, Anjum Hajat

**Affiliations:** 1 Department of Environmental and Occupational Health Sciences, University of Washington, Seattle, WA USA; 2 Department of Epidemiology, University of Washington, Seattle, WA USA; 3 Department of Epidemiology and Prevention, Wake Forest School of Medicine, Winston-Salem, NC USA; 4 Department of Medicine, University of Washington, Seattle, WA USA

**Keywords:** Neighborhood greenspace, Environmental epidemiology, Type 2 diabetes

## Abstract

**Background:**

Neighborhood greenspaces provide opportunities for increased physical activity and social interaction, and thus may reduce the risk of Type 2 diabetes. However, there is little robust research on greenspace and diabetes. In this study, we examine the longitudinal association between neighborhood greenspace and incident diabetes in the Multi-Ethnic Study of Atherosclerosis.

**Methods:**

A prospective cohort study (*N* = 6814; 2000-2018) was conducted to examine the association between greenspace, measured as annual and high vegetation season median greenness determined by satellite (Normalized Difference Vegetation Index) within 1000 m of participant homes, and incident diabetes assessed at clinician visits, defined as a fasting glucose level of at least 126 mg/dL, use of insulin or use of hypoglycemic medication, controlling for covariates in stages. Five thousand five hundred seventy-four participants free of prevalent diabetes at baseline were included in our analysis.

**Results:**

Over the study period, 886 (15.9%) participants developed diabetes. Adjusting for individual characteristics, individual and neighborhood-scale SES, additional neighborhood factors, and diabetes risk factors, we found a 21% decrease in the risk of developing diabetes per IQR increase in greenspace (HR: 0.79; 95% CI: 0.63, 0.99).

**Conclusions:**

Overall, neighborhood greenspace provides a protective influence in the development of diabetes, suggesting that neighborhood-level urban planning that supports access to greenspace--along with healthy behaviors--may aid in diabetes prevention. Additional research is needed to better understand how an area’s greenness influences diabetes risk, how to better characterize greenspace exposure and usage, and future studies should focus on robust adjustment for neighborhood-level confounders.

**Supplementary Information:**

The online version contains supplementary material available at 10.1186/s12940-021-00824-w.

## Background

Type 2 diabetes is increasingly common in the U.S. and worldwide, with the age-adjusted prevalence of diagnosed type 2 diabetes increasing from 6.4% in 1990-2002 to 9.4% in 2013-2016 in the U.S. [1]. Research has shed light on the role of diet, exercise, and adiposity in type 2 diabetes, and prior work has emphasized the importance of individual risk factors [2]. The role of environmental factors, particularly at the neighborhood scale, is less well understood. However, ongoing work indicates that an individual’s residential neighborhood may influence an individual’s risk [3, 4].

Increasingly, research has focused on the role of the larger neighborhood social and physical environments, including modifiable community-scale factors such as greenspace, in diabetes risk and prevention. For example, research has explored neighborhood deprivation and other measures of neighborhood socioeconomic status (SES), neighborhood walkability, neighborhood social support and social cohesion, neighborhood safety, and neighborhood greenspace as factors relevant to diabetes [3–6]. In particular, there is a growing body of research on the association between neighborhood greenspace and diabetes [7–16]. Greenspace is often conceptualized as the amount of or proximity to greenness or vegetation, which, in urban settings, includes parks, forests, fields, and outdoor recreation areas [5]. Greenspace is hypothesized to act through multiple mechanisms, including through increasing physical activity and reduced stress. However, the degree to which greenspace operates independently on diabetes is not well understood [3–6].

Most studies examining associations between greenspace and diabetes have used a cross-sectional study design [7–13]. Longitudinal analyses generally suggest an association between cumulative greenspace exposure and risk of diabetes, though there is variability in exposure assessment and results across these studies [14–16]. Cohort studies in the UK and the US identified an inverse relationship between greenspace and risk of diabetes, but are limited by self-reported outcome data or minimal racial and ethnic diversity [14, 15]. A study using national medical claims data in Taiwan observed this relationship in cross-sectional but not longitudinal analyses, though was limited in adjustment for potential confounders including time-varying and neighborhood-level confounders [16]. Our study is poised to fill these gaps by investigating the association between greenspace and incident diabetes in the U.S. in a well characterized cohort with clinician and lab-based diabetes diagnoses and longitudinal covariate data.

In this study, we examine the association between greenspace exposure and the development of incident diabetes in the Multi-Ethnic Study of Atherosclerosis (MESA). This study is the first longitudinal study examining greenspace exposure and incident diabetes in the U.S. with longitudinal covariate data.

## Methods

### Study population

This study was conducted with data from MESA and its ancillary studies. MESA is a prospective cohort assembled initially to understand the relationship between subclinical cardiovascular disease markers and the incidence of cardiovascular disease. At baseline, the MESA cohort comprised 6814 non-institutionalized adults who were cardiovascular disease-free, aged 45-84 years, who self-identified as white, black, Hispanic, or Chinese from 6 study sites: New York, NY, Baltimore, MD, Forsyth County, NC, Chicago, IL, St. Paul, MN, and Los Angeles, CA [17]. In this analysis, we further restricted the analytic sample to the 5574 participants who were diabetes free at baseline. The study included follow-up phone questionnaires every 9-12 months. The baseline exam occurred from July 2000 through August 2002, and five follow-up in-person exams occurred an average of 1.6, 3.2, 4.8, 9.5, and 15.8 years later. The cohort included 5413; 5184; 5080; 4155; and 2962 participants without diabetes at each of the follow-up exams, respectively. Informed consent was obtained from all participants, and the study was approved by the institutional review boards at each study site.

### Outcome

Type 2 diabetes status was ascertained at each follow-up clinical visit (exam) for study participants, and all participants with prevalent type 2 diabetes at baseline were excluded from this analysis. Using criteria from the American Diabetes Association [18], incident cases were defined as having a fasting glucose level of at least 126 mg/dL at the exam, use of insulin, or use of hypoglycemic medication. This study considered all incident cases as type 2 diabetes; however, these cases may include patients who developed Latent Autoimmune Diabetes in Adults (LADA). Additionally, because participants were evaluated for diabetes at exam intervals, the diabetes diagnosis dates were subject to interval censoring. Due to the uncertainty of the exact onset of diabetes, we assigned the date of diabetes diagnosis as the midpoint between the prior exam and the exam where the diagnosis was given.

### Exposure

Greenspace was characterized using the Normalized Difference Vegetation Index (NDVI), updated at each exam to match changes in residence. NDVI is a commonly used measure of greenspace derived from satellite imagery [19–21]. For our analysis, NDVI from 2006 satellite imagery with a 250 m resolution was considered on a scale from 0 to 255-pixel brightness [22]. On this scale, areas with dense vegetation have values around 200, and non-vegetative surfaces such as roads and buildings have values less than 50.

We obtained participant residence information from the MESA Neighborhood Ancillary study. Home addresses were obtained at baseline and during all follow-up phone calls during the study period. At each participant residence, we characterized greenspace within a varying radius (buffer) to capture greenspace in the participant’s residential neighborhood. For our primary analysis, we used the annual median NDVI within a 1 km radius as our a priori exposure, based on prior research indicating that a 1 km radial buffer had the highest correlation with self-described neighborhood for the corresponding spatial resolution of the NDVI data [21]. NDVI was linked to the address reported at each exam to account for differences in exposure across the study period due to a change in residential address. In sensitivity analyses, we examined annual median NDVI within 500 m and 2.5 km, as well as high vegetation season (April 1 – September 30) median NDVI within a 1 km radius. These two time periods were used because NDVI can vary dramatically based on season.

### Covariates

MESA ascertained important potential confounders through a combination of interviews, exams, and census data [17]. We included several individual sociodemographic characteristics: sex, age, race and ethnicity (non-Hispanic white, non-Hispanic black, Hispanic/Latinx and Chinese), education level (less than or equal to high school, some college, bachelor’s degree or higher), employment status (working outside the home or not) and income level. Participant income was calculated from the midpoint of total annual household income specified in categories divided by number of household members (<$12,000; $12,000-24,999; $25,000-39,999, $40,000-74,999, >$75,000). We also included several neighborhood-level characteristics to control for neighborhood-level confounding: neighborhood deprivation index (NDI) [3, 4], social cohesion [23–25], safety [26–28], walkability score [3, 6], and proportion urban all measured at the census-tract level. NDI is comprised of several variables from U.S. Census and American Community Survey data representing characteristics of education, occupation and employment, household income and wealth, poverty, and housing, and was assessed at exams 1, 3, and 4 [29–32]. Higher index values indicate greater neighborhood deprivation (i.e., lower neighborhood SES) [32]. Social cohesion, safety, and walkability scores were weighted averages by census tract based on survey responses from MESA participants at baseline and a random sample of people living in the same census tracts as MESA participants in 2004, as in prior MESA research [33]. Social cohesion was measured with a four-item scale including items about willingness to help neighbors, getting along with neighbors, neighborhood trust, and sharing values with neighbors. Safety was measured using a two-item scale about feeling safe in one’s neighborhood and violence in the neighborhood. Walkability was measured using a four-item scale about the perceived ease of walking to places in the neighborhood, seeing others walking or exercising, and pleasantness of walking in the neighborhood. Proportion urban measures the percentage of the census tract population living in an urban area, measured using U.S. Census data [30]. Finally, our primary model is additionally adjusted for study site due to the differing population characteristics and NDVI by site.

We additionally adjusted for type 2 diabetes risk factors including family history of diabetes, chronic stress, alcohol consumption, smoking status, body mass index (BMI), and physical activity score. Family history of diabetes was coded dichotomously from responses to a family medical history questionnaire answered at exam 2. Chronic stress was determined from the Chronic Burden scale, a six-item questionnaire given at exams 1 and 3 [34]. Participants were categorized as chronically stressed if they had indicated 6 or more months of strain or difficulties from a relationship, job, or finances, or 6 or more months of medical-related issues for themselves or someone close to them. As in prior MESA research [35], alcohol consumption was categorized as heavy, moderate, and not current drinker and smoking status was categorized as current, former, and never using data available at exams 1-6. BMI was assessed at exams 1-6. Physical activity score was assessed at exams 1-3 and 5, and is the sum of a reverse measure of sedentary behavior, in quartiles (0 = most sedentary, 3 = least sedentary) and a measure of moderate-vigorous physical activity (MVPA), in quartiles (1 = least MVPA, 4 = most MVPA), resulting in a physical activity score from 1 to 7 [36]. Additionally, several of the model covariates varied over each of the 6 exams in order to more accurately capture and control for possible confounding factors. These include: chronic stress, income, employment status, alcohol consumption, smoking status, BMI, physical activity score, and NDI. For all time-varying covariates, data were carried forward if they were missing from future follow-up exams.

### Statistical approach

We conducted a survival analysis examining the association between greenspace exposure and incident diabetes in the MESA cohort. We used Cox proportional hazards regression with time-varying covariates with calendar time as the time scale to estimate the hazard ratio (HR) of incident diabetes risk for an interquartile (IQR) increase in NDVI [35, 37]. Participants were considered in the risk set until diagnosis of diabetes, last follow-up visit, or at the end of the study period (exam 6), whichever occurred first. In a sensitivity analysis, we re-ran the models to estimate the HR for a 0.1 increase in NDVI, on a − 1 to 1 scale to compare with results in the literature [5, 38].

We determined confounders a priori, and they were added in stages. Model 1 adjusted for age, sex, race/ethnicity, study site, education level, income, employment status, neighborhood deprivation index, neighborhood social cohesion, walkability, safety, and percent urban. The primary model (Model 2) adjusted for the covariates in Model 1 as well as family history of diabetes, BMI, physical activity score, chronic stress, drinking, and smoking. We ran an additional model (Model 3) excluding all potential mediators (BMI, physical activity score, walkability, safety, neighborhood social cohesion) to assess any suggestion of mediation by these factors.

We hypothesized a priori that the association between NDVI and incident diabetes may vary importantly by site, based on the skewed nature of NDVI by site, varying urbanicity, and differing population characteristics in each site. To assess the adequacy of the adjustment for study site using a fixed effect, we conducted a sensitivity analysis using a stratified Cox model with stratification by MESA study site. A stratified Cox model allows the baseline hazard to differ by MESA study site.

We evaluated the assumption of proportional hazards by plotting and testing Schoenfeld residuals and assessing the trend over time. In sensitivity analyses, we examined additional NDVI exposures (500 m and 2.5 km buffers, and high vegetation season NDVI), and effect modification by urbanicity and by neighborhood deprivation index based on prior work showing evidence that the association between greenspace and diabetes is different for urban versus rural areas [14], and by measures of neighborhood socioeconomic status [7, 12, 14]. We also examined effect modification by sex to compare results with prior studies [11, 16].

## Results

Of the 5574 MESA study participants with no prevalent diabetes at baseline, 886 (15.9%) developed incident diabetes over the study period. Those developing incident diabetes were more likely to be Hispanic or Non-Hispanic Black, more likely to have a high school or GED degree or less, more likely to have lower incomes, and more likely to have a family history of diabetes than those who did not develop diabetes during the study period (Table 1).

**Table 1:** Participant characteristics at baseline by diabetes status

	No incident diabetes (***N*** = 4688)	Incident diabetes (***N*** = 886)	Overall (***N*** = 5574)
**Sex**			
Female	2530 (54.0%)	445 (50.2%)	2975 (53.4%)
Male	2158 (46.0%)	441 (49.8%)	2599 (46.6%)
**Age**			
45-54	1382 (29.5%)	314 (35.4%)	1696 (30.4%)
55-64	1265 (27.0%)	273 (30.8%)	1538 (27.6%)
65-74	1357 (28.9%)	228 (25.7%)	1585 (28.4%)
75-84	679 (14.5%)	70 (7.9%)	749 (13.4%)
Missing	5 (0.1%)	1 (0.1%)	6 (0.1%)
**Race/Ethnicity**			
Hispanic	905 (19.3%)	242 (27.3%)	1147 (20.6%)
Non-Hispanic Black	1182 (25.2%)	256 (28.9%)	1438 (25.8%)
Non-Hispanic Chinese	530 (11.3%)	114 (12.9%)	644 (11.6%)
Non-Hispanic White	2071 (44.2%)	274 (30.9%)	2345 (42.1%)
**Education**			
HS/GED or less	1535 (32.7%)	325 (36.7%)	1860 (33.4%)
Some college	1318 (28.1%)	277 (31.3%)	1595 (28.6%)
Bachelors or higher	1817 (38.8%)	283 (31.9%)	2100 (37.7%)
Missing	18 (0.4%)	1 (0.1%)	19 (0.3%)
**Income Category**			
< $12,000	1192 (25.4%)	257 (29.0%)	1449 (26.0%)
$12,000-$24,999	1301 (27.8%)	263 (29.7%)	1564 (28.1%)
$25,000-$39,999	926 (19.8%)	181 (20.4%)	1107 (19.9%)
$40,000-$74,999	927 (19.8%)	131 (14.8%)	1058 (19.0%)
$75,000+	143 (3.1%)	15 (1.7%)	158 (2.8%)
Missing	199 (4.2%)	39 (4.4%)	238 (4.3%)
**Employment**			
Employed	2333 (49.8%)	478 (54.0%)	2811 (50.4%)
Unemployed/retired	2337 (49.9%)	406 (45.8%)	2743 (49.2%)
Missing	18 (0.4%)	2 (0.2%)	20 (0.4%)
**Site**			
Forsyth County, NC	772 (16.5%)	137 (15.5%)	909 (16.3%)
New York City, NY	724 (15.4%)	174 (19.6%)	898 (16.1%)
Baltimore, MD	725 (15.5%)	130 (14.7%)	855 (15.3%)
St. Paul, MN	746 (15.9%)	136 (15.3%)	882 (15.8%)
Chicago, IL	881 (18.8%)	127 (14.3%)	1008 (18.1%)
Los Angeles, CA	840 (17.9%)	182 (20.5%)	1022 (18.3%)
**Family history of diabetes**			
Family history	1384 (29.5%)	388 (43.8%)	1772 (31.8%)
No family history	3119 (66.5%)	479 (54.1%)	3598 (64.5%)
Missing	185 (3.9%)	19 (2.1%)	204 (3.7%)
**Chronically stressed**			
Chronically stressed	2815 (60.0%)	529 (59.7%)	3344 (60.0%)
Not chronically stressed	1822 (38.9%)	354 (40.0%)	2176 (39.0%)
Missing	51 (1.1%)	3 (0.3%)	54 (1.0%)
**BMI**			
Mean (SD)	27.5 (5.0)	30.8 (5.7)	28.0 (5.3)
**Physical activity level**			
High	977 (20.8%)	168 (19.0%)	1145 (20.5%
Medium	2853 (60.9%)	546 (61.6%)	3399 (61.0%)
Low	843 (18.0%)	172 (19.4%)	1015 (18.2%)
Missing	15 (0.3%)	0 (0%)	15 (0.3%)
**Alcohol consumption**			
Heavy	726 (15.5%)	122 (13.8%)	848 (15.2%)
Moderate	2021 (43.1%)	377 (42.6%)	2398 (43.0%)
Never/not current	1909 (40.7%)	384 (43.3%)	2293 (41.1%)
Missing	32 (0.7%)	3 (0.3%)	35 (0.6%)
**Smoking**			
Current	686 (14.6%)	142 (16.0%)	828 (14.9%)
Former	1774 (37.8%)	325 (36.7%)	2099 (37.7%)
Never	2210 (47.1%)	418 (47.2%)	2628 (47.1%)
Missing	18 (0.4%)	1 (0.1%)	19 (0.3%)
**Neighborhood Deprivation Index (NDI) tertile**			
High SES (low NDI)	1543 (32.9%)	253 (28.6%)	1796 (32.2%)
Mid SES (mid NDI)	1497 (31.9%)	298 (33.6%)	1795 (32.2%)
Low SES (high NDI)	1470 (31.4%)	323 (36.5%)	1793 (32.2%)
Missing	178 (3.8%)	12 (1.4%)	190 (3.4%)
**Neighborhood Social Cohesion**			
High	1503 (32.1%)	283 (31.9%)	1786 (32.0%)
Mid	1510 (32.2%)	280 (31.6%)	1790 (32.1%)
Low	1486 (31.7%)	305 (34.4%)	1791 (32.1%)
Missing	189 (4.0%)	18 (2.0%)	207 (3.7%)
**Neighborhood Walkability**			
High	1532 (32.7%)	240 (27.1%)	1772 (31.8%)
Mid	1478 (31.5%)	298 (33.6%)	1776 (31.9%)
Low	1446 (30.8%)	330 (37.2%)	1776 (31.9%)
Missing	232 (4.9%)	18 (2.0%)	250 (4.5%)
**Neighborhood Safety**			
High	1505 (32.1%)	267 (30.1%)	1772 (31.8%)
Mid	1490 (31.8%)	286 (32.3%)	1776 (31.9%)
Low	1461 (31.2%)	315 (35.6%)	1776 (31.9%)
Missing	232 (4.9%)	18 (2.0%)	250 (4.5%)
**NDVI (Mean (SD))**			
Annual median 1000 m	127 (33.3)	129 (33.5)	127.5 (33.3)

Note: All participants are diabetes free at baseline. All characteristics are based on data collected at baseline

The distribution of NDVI is skewed by study site, with some sites having predominantly high NDVI and thus more vegetated landscapes, while others have a wider range of NDVI (Fig. S1). Additionally, the study-level IQR (55) is much larger than each of the site-specific IQRs (ranged between 10.75 and 44), indicating that the range of NDVI at each site is much smaller than in the study overall (Table S1).

Results from the primary Cox proportional hazards regression model (Model 2) indicate an inverse association between greenspace and incident diabetes for both 1 km annual (HR: 0.79 (95% CI: 0.63, 0.99)) and 1 km high vegetation season median NDVI (HR: 0.74 (95% CI: 0.57, 0.95)). For each IQR increase in NDVI, the risk of developing diabetes is 21% less among those with higher neighborhood NDVI compared to lower, controlling for individual characteristics, neighborhood-level covariates, and diabetes risk factors. In models with 500 m and 2.5 km NDVI buffers, we also find an inverse effect but confidence intervals include the null (Table 2). In models excluding potential mediators (Model 3), we find attenuated hazard ratios for all buffer radii (Table 2). When stratified by site using a stratified Cox model rather than adjusting for site with a fixed effect, the results are nearly identical (Table 3).

**Table 2:** Hazard ratios (HRs) and 95% confidence intervals (CIs) for incident diabetes corresponding to an interquartile range (IQR) increase in NDVI

Buffer size	HR (95% CI)
Model 1^a^	0.83 (0.66, 1.03)
Model 2^b^	0.79 (0.63, 0.99)*
Model 3^c^	0.88 (0.72, 1.08)
**1 km radius – high vegetation season median**	
Model 1^a^	0.79 (0.61, 1.01)
Model 2^b^	0.74 (0.57, 0.95)*
Model 3^c^	0.84 (0.67, 1.06)
**500 m radius - annual median**	
Model 1^a^	0.86 (0.69, 1.07)
Model 2^b^	0.84 (0.67, 1.05)
Model 3^c^	0.90 (0.73, 1.09)
**2.5 km radius - annual median**	
Model 1^a^	0.90 (0.7, 1.15)
Model 2^b^	0.84 (0.65, 1.08)
Model 3^c^	0.95 (0.76, 1.20)

^a^ Model 1 adjustment set: age, sex, race/ethnicity, education category, income category, employment status, neighborhood deprivation index, neighborhood social cohesion, neighborhood walkability, neighborhood safety, urbanicity, and site

^b^ Model 2 (primary model) adjustment set: Model 1 adjustment set plus family history of diabetes, BMI, physical activity score, chronic stress, smoking, drinking

^c^Model 3 adjustment set: site, age, sex race/ethnicity, education category, income category, employment status, neighborhood deprivation index, family history of diabetes, chronic stress, smoking, drinking

* *p* ≤ 0.05

**Table 3:** Hazard ratios (HRs) and 95% confidence intervals (CIs) for incident diabetes corresponding to an interquartile range (IQR) increase in NDVI from stratified Cox models, stratified by study site

Buffer size	HR (95% CI)
**1 km radius – annual median**	
Model 1^a^	0.83 (0.66, 1.03)
Model 2^b^	0.79 (0.63, 0.99)*
**1 km radius – high vegetation season median**	
Model 1^a^	0.79 (0.61, 1.01)
Model 2^b^	0.74 (0.57, 0.95)*
**500 m radius - annual median**	
Model 1^a^	0.86 (0.69, 1.06)
Model 2^b^	0.84 (0.67, 1.05)
**2.5 km radius - annual median**	
Model 1^a^	0.89 (0.70, 1.14)
Model 2^b^	0.84 (0.65, 1.08)

^a^ Model 1 adjustment set: age, sex, race/ethnicity, education category, income category, employment status, neighborhood deprivation index, neighborhood social cohesion, neighborhood walkability, neighborhood safety, urbanicity, and site

^b^ Model 2 (primary model) adjustment set: Model 1 adjustment set plus family history of diabetes, BMI, physical activity score, chronic stress, smoking, drinking

* *p* ≤ 0.05

In our model adjusted for individual and neighborhood characteristics with a 1 km annual NDVI buffer (Model 1), we find an inverse association between greenspace and incident diabetes, however, confidence intervals include the null (HR: 0.83; 95% CI: 0.66, 1.03) (Table 2). In models with differing buffers (Table 2), excluding potential mediators (Table 2), and with a stratified Cox model (Table 3), the results remain similar.

An analysis examining effect modification by NDI, a measure of neighborhood SES, found no evidence that the association between greenspace exposure and incident diabetes varies by NDI tertile, calculated across the entire study sample (Table 4). Furthermore, we find evidence of effect modification by sex, with female participants experiencing a more protective effect compared to male participants (Table 4). Additionally, we were unable to formally evaluate effect modification by urbanicity due to the highly skewed nature of our data, with nearly all participants living in urban areas.

**Table 4:** Results from multiplicative interaction by levels of neighborhood deprivation index (NDI) and by sex for the primary model (Model 2)

Model	1 km annual median NDVI	1 km high vegetation season median NDVI
**Model 2, plus interaction by NDI**^**a**^ **(*p*-value)**	0.64	0.58
**Model 2, plus interaction by sex**^**b**^ **(HR (95% CI))**	1.33 (1.05, 1.69)*	1.39 (1.08, 1.80)*

^a^Reported *p*-values for NDI are derived from an ANOVA comparing the interaction model to a model without an interaction

^b^ Reported coefficients for sex are derived from the interaction term of NDVI and sex, showing the effect of greenspace by sex. Females are the referent category

Adjustment set includes all Model 2 covariates plus interaction term between greenspace and NDI tertile or between greenspace and sex

* *p* ≤ 0.05

In a sensitivity analysis evaluating the risk of diabetes per 0.1 increase in NDVI, we find similar but attenuated hazard ratios. In our primary model (Model 2), for each 0.1 increase in NDVI, the risk of developing diabetes is 5% less among those with higher neighborhood NDVI compared to lower (HR: 0.95; (95% CI: 0.91, 0.99)) (Table S2).

## Discussion

Overall, we find a significant inverse association between greenspace and incident diabetes in the MESA cohort in our fully-adjusted model (Model 2). Additionally, we observe an attenuated inverse effect with a more parsimonious model (Model 1) and when using a 500 m or 2.5 km buffer radius for NDVI exposure, with confidence intervals that include the null. Our results hold when we use a stratified Cox model for study site (Table 3), and when we use an NDVI scale commonly used in the literature (Table 2). We find evidence of effect modification by sex, and no evidence of effect modification by neighborhood deprivation (Table 4). Although not the main objective of the paper, we find limited evidence of mediation by a combined set of mediators when using an exploratory mediation analysis approach.

Our results agree with the main findings in the literature that generally report inverse associations between greenspace and diabetes [7–16]. However, it is challenging to compare results across studies examining the association between greenspace and diabetes due to the varying measures of greenspace (proportion greenspace or park space within a buffer, distance to parks, NDVI), differing spatial scales, differing study designs, and vastly different study populations [5, 6, 20, 21, 26, 38, 39]. Three studies have reported results on the longitudinal association between greenspace and incident diabetes. Dalton et al. found a significant inverse association, with greenspace measured as proportion greenspace within an 800 m buffer determined from the UK Land Cover Map, comparing the highest to the lowest quartile, and incident diabetes ascertained through self-report (HR: 0.81; 95% CI: 0.67, 0.99). This study was based in the UK, with a larger cohort than MESA (> 23,000 participants), and a similar set of covariates [14]. Further, Lee et al. found a significant cross-sectional association between greenspace, as proportion greenspace by census block group comparing highest to lowest quartile, and prevalent diabetes, defined by fasting plasma glucose ≥126 mg/dL, but this association was null in their longitudinal analysis (OR: 0.70; 95% CI: 0.41, 1.19). Participants in this study were from the a cohort in Massachusetts, with a comparable sized cohort to MESA (~ 4000 participants), and similar covariate adjustment [15]. Finally, Tsai et al. found a significant inverse association, with greenspace reported per IQR increase in NDVI among a sample of individuals from Taiwan’s National Health Insurance medical claims data (HR: 0.81; 95% CI: 0.79-0.82). This cohort was substantially larger than MESA (> 400,000), with a more limited adjustment set due to the lack of neighborhood-level covariate data [16].

Several other studies have examined the cross-sectional association between measures of greenspace and prevalent diabetes [7–13]. Many found significant inverse associations, however, in some studies, the strength of association was very small [10], or found in only one of several measures of greenspace [8]. Furthermore, among these cross-sectional studies, only two used NDVI as the measure of greenspace, and both reported per 0.1 unit increase in NDVI (on a − 1 to 1 scale), with a 500 m buffer [13] or at the block-level [12]. Moreover, only three studies identified diabetes through blood samples [9, 13, 15], while four used diagnosis codes or claims data [8, 10, 12, 16], and three relied on self-reported diagnosis [7, 11, 14]. Thus, it is challenging to directly compare our results and to put our results into broader context.

Our results indicate possible confounding by neighborhood-level factors, including by measures of neighborhood SES. Several studies provide evidence of differential exposure to greenspace by neighborhood SES [3–5, 40]. In particular, individual neighborhood selection is a common methodological issue, and if not adequately controlled for, can lead to biased estimates [3, 4, 41, 42]. In a study where individuals were sampled from the MESA study site locations, authors reported individuals had different residential preferences based on their SES and individual characteristics, indicating that residential self-selection is a critical confounder [42]. In an effort to address this, we adjusted for neighborhood-level proxy measures (social cohesion, safety, walkability) that may account for some of the residual neighborhood-level confounding and residential self-selection not accounted for by adjustment for neighborhood deprivation index. Future studies should collect and adjust for individual residential preferences in order to account for this important source of bias.

The current literature indicates several possible mechanisms through which greenspace may reduce the risk of diabetes [3, 5, 6, 26, 38, 39]. One proposed mechanism suggests that access to greenspace increases physical activity, particularly in urban environments, leading to lower BMI and adiposity, which are associated with lower rates of several chronic conditions, including diabetes [3–5]. However, existing evidence for mediation by measures of physical activity and adiposity is mixed [13, 14]. Other proposed mechanisms include reduced stress, increased social cohesion, and reduction in air and noise pollution, and have not been fully explored for diabetes [3, 5, 6, 13, 23]. Results from Model 3 in this analysis suggest possible mediation by a combination of neighborhood (social cohesion, safety, walkability) and individual (BMI, physical activity) factors. Further mediation analyses are needed to increase the understanding of possible mechanisms. However, methods for mediation analysis with survival outcomes and time-varying mediators need further development [43].

The primary strength of this study is that, in leveraging the MESA cohort, we are able to take advantage of the study’s rich longitudinal covariate data, allowing for several time-varying covariates, which are not present in the three other longitudinal studies on greenspace exposure and incident diabetes [14–16]. Furthermore, this study used blood measurements from in-person clinic visits and medication data to ascertain diabetes diagnoses, while previous studies examining greenspace and diabetes often rely on self-reported diabetes diagnosis [7, 11, 14] or administrative data [8, 10, 12, 16]. Additionally, this cohort is both racially and ethnically diverse, and represents several regions in the U.S. We also examined the association using different exposure buffers to understand how the association may vary with different neighborhood sizes [21]. Finally, we included several neighborhood-level covariates in an attempt to better control for neighborhood SES and neighborhood-level confounding; many of these covariates are not typically included in other studies [7–16].

There are several limitations of this work that can be improved upon in future research. NDVI is not a specific measure of greenspace and has no information about the quality of greenspaces. While NDVI is a standard measure of greenspace, a higher spatial and temporal resolution of NDVI satellite data sources, or alternate assessments of greenspace exposure may aid in reducing exposure misclassification [3, 20, 21]. Further, while this study has a wealth of longitudinal covariate data, there is no data regarding greenspace usage. Future work should consider alternate definitions of greenspace and take into account greenspace usage and accessibility rather than solely neighborhood vegetation. Additionally, we did not formally account for interval censoring, which may bias our results.

## Conclusions

Overall, we find a significant inverse association from our fully-adjusted model between greenspace exposure and incident diabetes and a non-significant inverse effect found in a more parsimonious model and when using differing neighborhood buffers. We additionally find evidence of moderation by sex, but no evidence of moderation by neighborhood deprivation index. These results indicate, as other studies have found, that the relationship between greenspace and diabetes is complex, and is likely confounded by several individual and neighborhood-level factors. Further work should consider alternate measures of greenspace that take into account greenspace usage, as well as more robust data collection and adjustment for individual and neighborhood SES, individual neighborhood preferences, and other neighborhood-level covariates. This study contributes to the literature suggesting that neighborhood greenspace is modestly protective against diabetes [7–16], indicating that neighborhood-level planning that supports healthy behaviors and lifestyles may aid in diabetes prevention.

## Supplementary Information

**Additional file 1.**

## Data Availability

The datasets analyzed during this study are not publicly available due to MESA study requirements.
