# Correction to: Neighborhood greenspace and risk of type 2 diabetes in a prospective cohort: the Multi-Ethnic Study of Atherosclerosis

**DOI:** 10.1186/s12940-022-00845-z

**Published:** 2022-03-03

**Authors:** Annie Doubleday, Catherine J. Knott, Marnie F. Hazlehurst, Alain G. Bertoni, Joel D. Kaufman, Anjum Hajat

**Affiliations:** 1 Department of Environmental and Occupational Health Sciences, University of Washington, Seattle, WA USA; 2 Department of Epidemiology, University of Washington, Seattle, WA USA; 3 Department of Epidemiology and Prevention, Wake Forest School of Medicine, Winston-Salem, NC USA; 4 Department of Medicine, University of Washington, Seattle, WA USA

**Correction to: Environ Health 21, 18 (2022)**

**https://doi.org/10.1186/s12940-021-00824-w**

Following the publication of the original article [1], the author reported an incorrect word in the Article title.

The title "Neighborhood greenspace and risk of type 2 diabetes in a prospective cohort: the **Multi-Ethncity** Study of Atherosclerosis"

should be "Neighborhood greenspace and risk of type 2 diabetes in a prospective cohort: the **Multi-Ethnic** Study of Atherosclerosis"

The original article has been updated
