# Supplementary material for: Neighborhood greenspace and risk of type 2 diabetes in a prospective cohort: the Multi-Ethncity Study of Atherosclerosis
Source: Environ Health. 2022 Jan 16;21:18. doi: 10.1186/s12940-021-00824-w (PMC8762964; doi:10.1186/s12940-021-00824-w)
Supplement: Supplementary file 1 — Additional file 1. [file 12940_2021_824_MOESM1_ESM.docx]

**Supplemental Figure S1.** Distribution of NDVI by site in the MESA cohort study participants


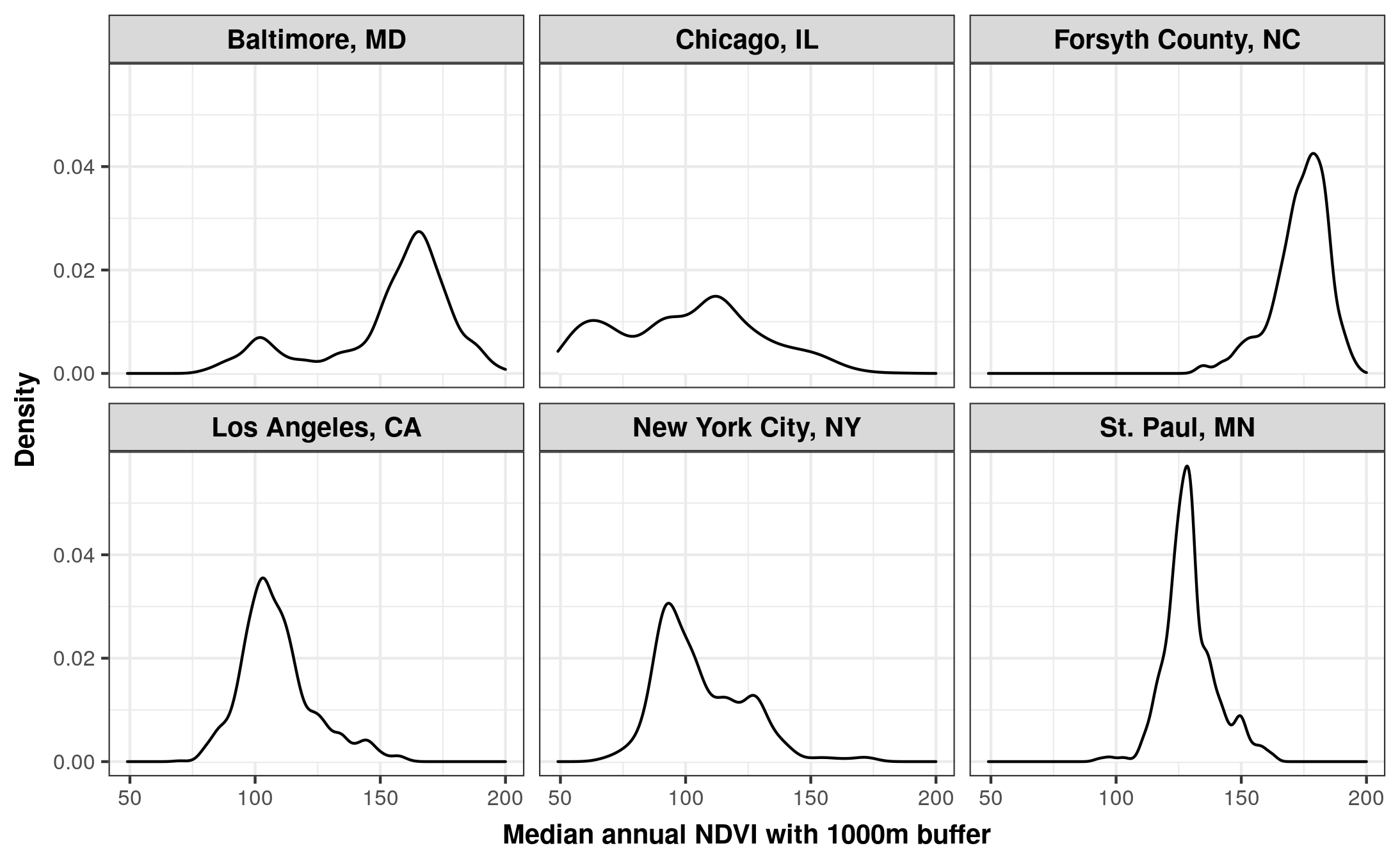


**Supplemental Table S1.** Distribution of NDVI by site and overall

| **Site** | **Min** | **25th %** | **Median** | **75th %** | **Max** | **IQR** |
| --- | --- | --- | --- | --- | --- | --- |
| **Annual Median NDVI, 1000m buffer** | | | | | | |
| All | 49 | 102 | 124 | 157 | 200 | 55 |
| Forsyth County, NC | 132 | 168 | 176 | 181 | 196 | 13 |
| New York, NY | 65 | 92 | 102 | 118 | 174 | 26 |
| Baltimore, MD | 80 | 147 | 162 | 170 | 200 | 23 |
| St. Paul, MN | 92 | 123.25 | 128 | 134 | 163 | 10.75 |
| Chicago, IL | 49 | 75 | 102 | 119 | 185 | 44 |
| Los Angeles, CA | 70 | 100 | 106 | 115 | 161 | 15 |
| **High Vegetation Season NDVI, 1000m buffer** | | | | | | |
| All | 60 | 106 | 140 | 178 | 218 | 72 |
| Forsyth County, NC | 144 | 186 | 196 | 202 | 216 | 16 |
| New York, NY | 75 | 100 | 111 | 128 | 218 | 28 |
| Baltimore, MD | 85 | 157 | 177 | 187 | 213 | 30 |
| St. Paul, MN | 98 | 153 | 161 | 169 | 210 | 16 |
| Chicago, IL | 60 | 82 | 116 | 134 | 204 | 52 |
| Los Angeles, CA | 70 | 99 | 106 | 115 | 156 | 16 |

Note: NDVI is on a scale from 0 to 255 in the MESA study.

**Supplemental Table S2.** Hazard ratios (HRs) and 95% confidence intervals (CIs) for incident diabetes corresponding to a 0.1 increase in NDVI (on a -1 to 1 scale)

| **Category** | **HR (95% CI)** |
| --- | --- |
| **1km radius – annual median** |  |
| Model 1^1^ | 0.96 (0.91, 1.01) |
| Model 2^2^ | 0.95 (0.90, 1.00) |
| Model 3^3^ | 0.97 (0.93, 1.02) |
| **1km radius – high vegetation season median** |  |
| Model 1^1^ | 0.96 (0.92, 1.00) |
| Model 2^2^ | 0.95 (0.91, 0.99)* |
| Model 3^3^ | 0.97 (0.93, 1.01) |
| **500m radius - annual median** | |
| Model 1^1^ | 0.96 (0.92, 1.01) |
| Model 2^2^ | 0.96 (0.91. 1.01) |
| Model 3^3^ | 0.98 (0.93, 1.02) |
| **2.5km radius - annual median** | |
| Model 1^1^ | 0.97 (0.92, 1.03) |
| Model 2^2^ | 0.96 (0.91, 1.02) |
| Model 3^3^ | 0.99 (0.94, 1.04) |

^1^ Model 1 adjustment set: age, sex, race/ethnicity, education category, income category, employment status, neighborhood deprivation index, neighborhood social cohesion, neighborhood walkability, neighborhood safety, urbanicity, and site

^2^ Model 2 (primary model) adjustment set: Model 1 adjustment set plus family history of diabetes, BMI, physical activity score, chronic stress, smoking, drinking

^3^Model 3 adjustment set: site, age, sex, race/ethnicity, education category, income category, employment status, neighborhood deprivation index, family history of diabetes, chronic stress, smoking, drinking.

* p ≤ 0.05
